# Mastering your fellowship: Part 3, 2022

**DOI:** 10.4102/safp.v64i1.5548

**Published:** 2022-06-13

**Authors:** Klaus B. von Pressentin, Mergan Naidoo, Andrew Ross, Ts’epo Motsohi, Tasleem Ras

**Affiliations:** 1 Division of Family Medicine, School of Public Health and Family Medicine, Faculty of Health Sciences, University of Cape Town, Cape Town, South Africa; 2 Department of Family Medicine, College of Health Sciences, University of KwaZulu-Natal, Durban, South Africa; 3 Division of Family Medicine and Primary Care, Department of Family and Emergency Medicine, Faculty of Medicine and Health Sciences, Stellenbosch University, Cape Town, South Africa

**Keywords:** family physicians, FCFP (SA) examination, family medicine registrars, postgraduate training, national exit examination, infectious diseases

## Abstract

The series, ‘Mastering your Fellowship’, provides examples of the question formats encountered in the written and clinical examinations, Part A of the Fellowship of the College of Family Physicians of South Africa (FCFP SA) examination. The series is aimed at helping family medicine registrars (and their supervisors) prepare for this examination.

## Introduction

This section in the *South African Family Practice* journal is aimed at helping registrars prepare for the Fellowship of the College of Family Physicians (FCFP SA) Final Part A examination and will provide examples of the question formats encountered in the written examination: multiple choice question (MCQ) in the form of single best answer (SBA – Type A) and extended matching question (EMQ – Type R); short answer question (SAQ), questions based on the critical reading of a journal article (CRJ: evidence-based medicine) and an example of an objectively structured clinical examination (OSCE) question. Each of these question types is presented based on the College of Family Physicians blueprint and the key learning outcomes of the FCFP (SA) programme. The MCQs are based on the 10 clinical domains of family medicine, the SAQs are aligned with the five national unit standards and the critical reading section will include evidence-based medicine and primary care research methods.

This month’s edition is based on unit standard 1 (effectively manage himself or herself, his or her team and his or her practice, in any sector, with visionary leadership and self-awareness to ensure the provision of high-quality, evidence-based care), unit standard two (evaluate and manage patients with both undifferentiated and more specific problems cost effectively according to the biopsychosocial approach), and unit standard 5 (conduct all aspects of healthcare in an ethical and professional manner). The domain covered in this edition is dermatology. The authors suggest that you attempt answering the questions (by yourself or with peers or supervisors) before finding the model answers online: http://www.safpj.co.za/.

Please visit the Colleges of Medicine website for guidelines on the Fellowship examination: https://www.cmsa.co.za/view_exam.aspx?QualificationID=9.

The authors are keen to hear about how this series is assisting registrars and their supervisors in preparing for the FCFP (SA) examination. Please email (editor@safpj.co.za) with your feedback and suggestions.

## Multiple choice question (MCQ): Single best answer

**FIGURE 1:**
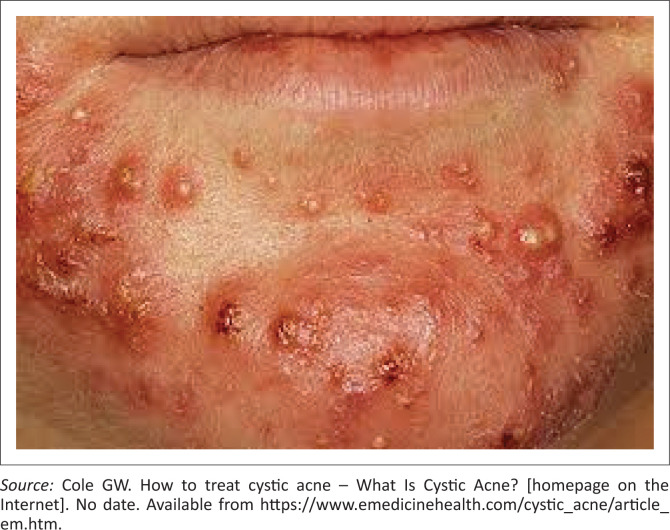
Multiple choice question clinical scenario.

A 22-year-old female presents to your district hospital. She is very concerned and ashamed about her face (see Figure 1). She states that this problem has been troubling her for three years. She asks for your advice to help manage her illness. What is the most appropriate next step in the management of her condition?

- Advise her about skin hygiene.
- Advise her on dietary interventions.
- Advise her to avoid sun exposure.
- Discourage her from squeezing the lesions.
- Obtain a sexual history from her.

*Answer:* e)

Model answer:

*Note*: *This MCQ was used in a past examination and often yielded poor results.*

The condition is one of severe acne vulgaris: an inflammatory disease of the hair follicle. It is caused by hormones and sebum gland keratinisation leading to follicular plugging, comedones and proliferation of *Propionibacterium*. Acne is divided into mild, moderate and severe forms. Mild acne consists of non-inflammatory comedones and moderate acne: a mixture of non-inflammatory comedones and inflammatory papules and pustules. Severe acne presents with widespread nodules and cysts and a preponderance of inflammatory papules and pustules.

Once diagnosed, general advice includes not squeezing the lesions, avoiding oily and greasy cosmetics and avoiding excessive facial washing. Mild acne is treated with topical preparations benzyl benzoate or topical retinoids. When using topical retinoids, it is important to advise on pregnancy and caution against sun exposure. Moderate acne is treated with topical preparations and oral doxycycline.

There is evidence of cystic acne with multiple inflamed cysts and nodules in this case. Once the diagnosis of severe acne is made, one needs to obtain a sexual history to exclude a pregnancy state. One also needs to get a history of venous thromboembolic disease and a family/personal history of breast carcinoma. The drug of choice is cyproterone acetate 2 mg plus ethinyl estradiol 35 μg taken orally. This drug can also serve as an oral contraceptive and is known by trade names such as Adco-Fem 35, Claro 35, Diane-35 and so on. The combination provides an antiandrogen and oestrogen preparation and is also used for hirsutism and alopecia. The drug is administered on the first day of the menstrual cycle, and an additional non-hormone method of contraception should be provided in the first 14 days of the cycle. One would continue the drug for three to four cycles after the signs subside. Cyproterone acetate is contraindicated in pregnancy and during breastfeeding. If pregnancy occurs during treatment, the drug must be stopped. The drug has other contraindications and cautions and prescribers should consult an appropriate pharmacological resource when prescribing the medication.

Patients who fail to respond to this treatment should be referred to a dermatologist. Another option for severe acne is oral isotretinoin. This drug also has implications for the pregnancy state, so one also needs to obtain a comprehensive sexual history when considering this drug.

### Further reading

- South African Department of Health (2019). Hospital-Level Standard Treatment Guidelines and Essential Medicines List. Pretoria, South African National Department of Health. (Available through the EMGuidance app on iPhone operating system (IOS) or Android.)

## Short answer question (SAQ): The family physician’s role as a care provider and consultant within the domain of infectious diseases

**FIGURE 2:**
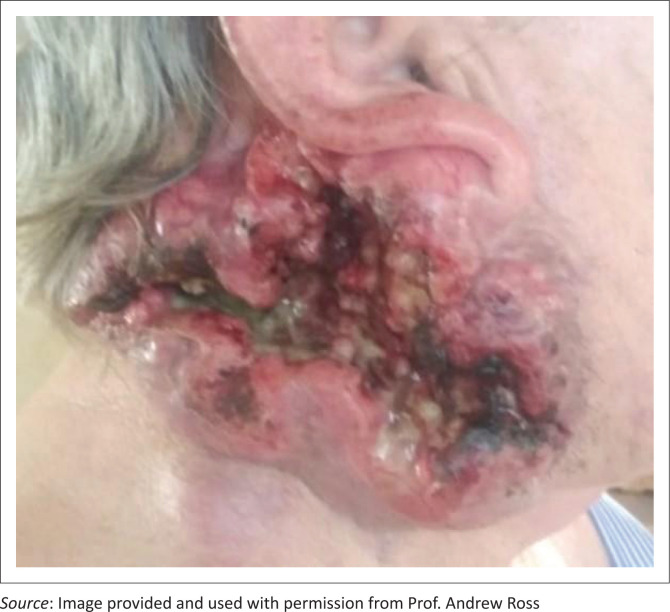
Short answer question clinical scenario.

A 52-year-old male patient has a necrotic ulcer involving the right parotid gland (see Figure 2). Histology shows that it is a basaloid squamous carcinoma. He has been to the oncology unit at the regional hospital, and they have told him that ‘there is no more that can be done’. The family is frantic as they are unable to control the bleeding from the ulcer and they have come to see you.

1. What are your treatment options for managing the bleeding (a) immediately and (b) in the long term? (5 marks)
2. How might the four ethical principles apply in this case? Justify your answer. (4 marks)
3. The patient decides to make a living will. What are the main objectives of a living will? (4 marks)
4. The patient complains of unbearable pain from the tumour. He is already on oral morphine 10 mg 8-hourly as needed. What are your options to adjust his pain medication? (5 marks)
5. The patient knows that there is no cure, and he feels that he is a burden to his family. He asks who could help him to end his life. How would you respond? (2 marks)

Total: 20 marks

Model answers

1. **What are your treatment options for managing the bleeding (a) immediately and (b) in the long term? (5 marks)**
  Immediate treatment:
  - Pack with gauze soaked in adrenalin. (1 mark)
  - Consider using tranexamic acid (Cyklokapron), either orally or sprinkled on the ulcer. (1 mark)
  - Consider haemostatic dressings such as calcium algicide (Kaltostat). (1 mark)
  - Apply a topical antibiotic to reduce bleeding from an infected wound. (1 mark)
  Longer term:
  - Specifically consider radiotherapy. (1 mark)
2. **How might the four ethical principles apply in this case? Justify your answer. (4 marks)**
  - Autonomy: Advanced directives, truth-telling and informed consent. (1 mark)
  - Non-maleficence: Do no psychological or emotional harm by destroying hope. Hope, although not vested in the cure, lies in holistic care for a peaceful death to achieve total pain control. (1 mark)
  - Beneficence: Give hope by assisting in a peaceful death. Manage all symptoms holistically. Break bad news by using a protocol. (1 mark)
  - Justice: Everyone deserves equal access to treatment. (1)
3. **The patient decides to make a living will. What are the main objectives of a living will? (4 marks)**
  A living will:
  - Provides instructions about a person’s wishes regarding artificial life support. (1 mark)
  - Can provide instructions about a person’s wishes regarding other forms of treatment or medical care to sustain his or her life, for example, hydration, feeding, analgesia and antibiotics. (1 mark)
  - Protects the patient’s rights and their autonomy. (1 mark)
  - Removes the burden of decision-making from family, friends and physicians. (1 mark)
4. **The patient complains of unbearable pain from the tumour. He is already on oral morphine 10 mg 8-hourly as needed. What are your options to adjust his pain medication? (5 marks)**
  - If there is nociceptive somatic or bone pain, make sure that he is on paracetamol 500 mg or 1 g, 6-hourly orally. (1 mark)
  - One may consider including a non-steroidal anti-inflammatory agent (e.g. Cox II inhibitor), but must be mindful that this may exacerbate the risk of bleeding. (1 mark)
  - For nociceptive visceral pain, give morphine 10 mg 4-hourly orally with 5 mg rescue doses if required rather than *ad hoc* as the scenario suggests. (1 mark)
  - Consider adding an antidepressant for depression and neuropathic pain if present. (1 mark)
  - Organise psychosocial (given the unsightly wound, etc.) and spiritual support to facilitate total pain control. (1 mark)
5. **The patient knows that there is no cure, and he feels that he is a burden to his family. He asks who could help him to end his life. How would you respond? (2 marks)**
  - Assisted suicide is illegal and no one in South Africa is allowed to help him to kill himself. (1 mark)
  - It would be important to give him hope by discussing and educating him about his physical, psychological and spiritual problems and management thereof. Arrange a family meeting, if he is agreeable, to talk about his feelings and their role in supporting him and to consider antidepressants, psychological and spiritual therapy. (1 mark)

### Further reading

- Bull A, Mash B. Advance directives or living wills: Reflections of general practitioners and frail care coordinators in a small town in KwaZulu-Natal. S Afr Fam Pract. 2012;54(6):507–512. https://doi.org/10.1080/20786204.2012.10874284
- Van Rooyen M, Hugo J, Louw M. Chapter 2.5.2: The consultation – The comprehensive three-stage assessment. In: Mash B, editor. Handbook of family medicine. 4th ed. Cape Town: Oxford University Press, 2017; p. 51–53.
- Gwyther L. Chapter 4.6.6: Palliative care. In: Mash B, editor. Handbook of family medicine. 4th ed. Cape Town: Oxford University Press, 2017; p. 129–132.
- Moodley K. Chapter 10.8: Family medicine ethics – The four principles of medical ethics. In Mash B, editor. Handbook of family medicine. 4th ed. Cape Town: Oxford University Press, 2017; p. 418–422.

## Critical appraisal of quantitative research

Read the accompanying article carefully and then answer the following questions (*total 30 marks*). As far as possible use your own words. Do not copy out chunks from the article. Be guided by the allocation of marks concerning the length of your responses.

- Meintjes KF. Primary health care nurses’ experiences of treating children with atopic eczema in Gauteng, South Africa. J Community Med Prim Health Care. 2018;30(2):1–11.

Total: 35 marks

1. Did the study address a clearly focused question? Discuss. (2 marks)
2. Identify three distinct arguments made by the author to justify and provide a rationale for the study. (3 marks)
3. Explain why a qualitative research methodology may be most appropriate for this research question. Comment on where and how a quantitative data collection methodology might still be applicable. (3 marks)
4. Were the participants well described? Justify your response. (3 marks)
5. Comment on the comprehensiveness and appropriateness of the data collection and analysis. (6 marks)
6. Appraise the reflexivity of the study (researcher positionality). (3 marks)
7. Comment on the reporting of the findings. In other words, were the descriptions of the findings clear and easy to understand? (3 marks)
8. Were the researchers’ conclusions appropriate and consistent with the data? Comment on the acceptability of their conclusions. (6 marks)
9. Use a structured approach (e.g. READER) to discuss the value of these findings to your practice. (6 marks)

Model answers:

1. **Did the study address a clearly focused question? Discuss. (2 marks)**
  - The author aimed to explore and describe primary health care (PHC) nurses’ experiences of managing children with atopic eczema in a district of Gauteng.
  - The question is focused as it describes the population of interest (PHC nurses), the condition or phenomenon of interest (these nurses’ management of children with atopic eczema) in a particular community or area (a district in the Gauteng province).
2. **Identify three distinct arguments made by the author to justify and provide a rationale for the study. (3 marks)**
  - The author refers to the significant burden of illness of atopic eczema globally and its detrimental effects on the psychology of those who suffer from it.
  - They refer to evidence in the literature that atopic eczema is commonly not treated appropriately, particularly in primary care, thereby contributing to the burden of illness.
  - As a result, they argue that understanding why atopic eczema is so poorly managed in primary care begins with exploring PHC nurses’ experience with treating it.
3. **Explain why a qualitative research methodology may be most appropriate for this research question. Comment on where and how a quantitative data collection methodology might still be applicable. (3 marks)**
  - Given that there has not been any previous research on nurse practitioners’ experiences in this area, an exploratory approach using qualitative research methods is appropriate. This will allow for a more in-depth exploration of data, particularly as it pertains to the nurse practitioners’ lived experiences.
  - Qualitative data are less restricted to discrete questions which might limit the respondent from expressing aspects of their experiences that were not considered during the construction of the questionnaire. This can occur when an area about which little is known is being explored.
  - The study was purposefully focused upon a few nurses who had extensive experience with the phenomenon. Therefore, the themes from their responses could be used to generate a quantitative data collection tool that could then be used to collect data from a larger population of primary care nurses, perhaps even beyond the Gauteng province. This would enrich and expand the study to a sequential mixed-methods study where the first phase is exploratory and the second phase is a quantitative explanatory one.
4. **Were the participants well described? Justify your response. (3 marks)**
  - The participants were described reasonably well because the inclusion criteria were clear. They specifically must have treated and referred children (aged 0–12 years) with atopic eczema.
  - They were also described as being based at any one of the 116 PHC facilities in Gauteng province. Their gender, type of clinic (municipal or provincial) and years of experience were well described. However, the researcher states that the nurses must have treated and referred children to the paediatric outpatient department. In addition, the name of the referral hospital is not stated.
  - Lastly, the researcher then describes the recruitment of nurses as being based on those who referred to the hospital outpatients department. There is no indication of how those who treated children without referring were recruited. This is one area that is unclear in the description of the participants.
5. **Comment on the comprehensiveness and appropriateness of the data collection and analysis. (6 marks)**
  - Congruity between the research methodology and the methods used to *collect data*: are the data collection methods appropriate to the methodology?
    ■ Yes, the study pursued a phenomenological approach and data were collected through phenomenological in-depth individual interviews (four participants) and focus group discussions (14 participants in three different focus groups). In phenomenology, the researcher seeks to elicit rich descriptions of the experience of a phenomenon, which aligns with the data collection methods.
    ■ The question asked during the interview was ‘how is it for you to treat children with atopic eczema’? Communication techniques were applied to confirm understanding of what the participants were saying. Data were collected until data saturation occurred; that is, no new information emerged.
  - Congruity between the research methodology and the *representation and analysis of data*: are the data analysed and represented in ways that are congruent with the stated methodological position?
    ■ The researcher described the first step of data analysis as to transcribe all the interviews verbatim. Subsequently, the researcher and an independent coder both used Tesch’s eight steps of the descriptive method of qualitative data analysis. Measures of trustworthiness and enhancement of credibility were considered in the analysis, including prolonged engagement with the participants and triangulation of data collection tools (focus groups, in-depth individual interviews and field notes, as well as member checking).
    ■ This method of data analysis is congruent with the stated methodological position, as the researcher stated that the study pursued a phenomenological approach to explore to gain a new and holistic understanding of the PHC management of children 0–12 years suffering from atopic eczema, in the public healthcare sector of a district of Gauteng, by asking the nurse participants to describe their experiences of treating children with atopic eczema. Congruity is demonstrated because the text generated from asking these questions is searched to establish the meaning of treating children with atopic eczema, and the meanings of all 18 participants were included in the report findings.
6. **Appraise the reflexivity of the study (researcher positionality). (3 marks)**
  - The study does not have a reflexivity report of any kind. The researcher does not describe her background, place of work or experience on the topic. From the title and author description, there is also no indication of her qualifications or profession. This makes it difficult to locate the researcher culturally or theoretically.
  - One can surmise that perhaps she is based in the hospital to which the referrals were made, because this is where the referring nurses seem to have been identified for recruitment. If this is the case, then her interviewing nurses who referred to the hospital is an important aspect of positionality upon which to reflect. This is because there tends to be an intimidating relationship between referral centres and the primary care centres that refer to them.
  - If she is a doctor, this is also an added dynamic given the historical power relationships between nurses and doctors. Similarly, if she is a nurse, there are other dynamics to consider and upon which to reflect.
7. **Comment on the reporting of the findings. In other words, were the descriptions of the findings clear and easy to understand? (3 marks)**
  - The presentation of results will depend on the level of analysis. In this study, a series of five themes related to the management challenges experienced by PHC nurses were reported, namely assessment and diagnosis challenges; knowledge levels; drug management and limited treatment protocols; health education and an ineffective referral system.
  - Each of these themes is supported by quotations from the raw data that speaks to the trustworthiness of the analysis. The participant’s voice behind each quotation is identified in brackets, without compromising confidentiality and anonymity.
  - The range of sources by data collection method (focus group or individual interview) and the number of supporting quotations make it easy for the reader to understand the link between the participants’ views and the key themes identified to describe the phenomenon of interest.
8. **Were the researchers’ conclusions appropriate and consistent with the data? Comment on the acceptability of their conclusions. (6 marks)**
  - When appraising an article, the reader should seek to satisfy himself or herself that the conclusions drawn by the research are based on the data collected and therefore are trustworthy. The criteria for trustworthiness (verification) are credibility (for internal validity), transferability (external validity), dependability (reliability) and confirmability (objectivity).
  - The author confirmed credibility by stating that there was prolonged engagement with the participants, triangulation and member checks.
  - Transferability was confirmed by providing a thick description of the PHC study context in the district and the research methodology and study’s findings, with a sufficient range of quotations from the participants. Furthermore, purposive sampling was used to identify information-rich participants with lived experiences of the phenomenon.
  - Dependability was ensured by creating an audit trail and triangulation.
  - Similarly, confirmability was ensured by triangulation. However, the reflexivity of the researcher was not stated.
  - Study limitations were not considered in the discussion section.
9. **Use a structured approach (e.g. READER) to discuss the value of these findings to your practice. (6 marks)**
  The READER format may be used to answer this question:
  - Relevance to family medicine and primary care?
  - Education – does it challenge existing knowledge or thinking?
  - Applicability – are the results applicable to my practice?
  - Discrimination – is the study scientifically valid enough?
  - Evaluation – given these facts, how would I score or evaluate the usefulness of this study to my practice?
  - Reaction – what will I do with the study findings?

The answer may be a subjective response but should be one that demonstrates a reflection on the possible changes within the student’s practise within the South African public healthcare system. It is acceptable for the student to suggest how their practice might change, within other scenarios after graduation (e.g. private general practice). The reflection on whether all important outcomes were considered is, therefore, dependent on the reader’s perspective (is there other information you would have liked to see?).

A model answer could be written from the perspective of the family physician employed in the South African district health system:

- R: This study is relevant to the African primary care context, as children living with atopic eczema are a common phenomenon, and there is a need to empower PHC nurses to manage these children, as nurses form the backbone of the PHC system by providing most of the first-contact care.
- E: The author motivates for increased support for the PHC nurses in managing these children with atopic eczema, as the study’s findings illustrate the challenges experienced.
- A: In this study, it would be possible to generalise the study’s findings to the wider South African setting, especially for the urban context with access to specialised referral services.
- D: In terms of discrimination, there is congruity between the research methodology and the *data collection methods,* as well as the *representation and analysis of data*. The criteria for trustworthiness (verification) are mostly met, except for the lack of stated reflexivity on the part of the researcher, which impacts the reader’s assessment of confirmability (objectivity).
- E: The study’s findings may be relevant to consider when planning coordination of care for children living with atopic eczema who are reliant on PHC nurses to manage and coordinate their care between primary and specialist services. It may be useful to remind oneself of the study limitations, which were not mentioned by the author, as well as that the study setting was that of an urban environment.
- R: The study findings may be useful to consider when reviewing quality improvement and in-service training opportunities for the primary care team in partnership with the specialist dermatology and allergology services.

### Further reading

- Williams V, Boylan AM, Nunan D. Critical appraisal of qualitative research: necessity, partialities and the issue of bias. BMJ Evid Based Med. 2020;25(1):9–11. https://doi.org/10.1136/bmjebm-2018-111132
- Tong A, Sainsbury P, Craig J. Consolidated criteria for reporting qualitative research (COREQ): A 32-item checklist for interviews and focus groups. Int J Qual Health Care. 2007;19(6):349–357. https://doi.org/10.1093/intqhc/mzm042
- Mabuza LH, Govender I, Ogunbanjo GA, Mash B. African primary care research: Qualitative data analysis and writing results. Afr J Prim Health Care Fam Med. 2014;6(1):1–5. https://doi.org/10.4102/phcfm.v6i1.640
- The Joanna Briggs Institute. JBI QARI critical appraisal checklist for interpretive & critical research. Adelaide: The Joanna Briggs Institute; 2014 [cited 2022 Mar 22]. Available from: https://jbi.global/sites/default/files/2019-05/JBI_Critical_Appraisal-Checklist_for_Qualitative_Research2017_0.pdf
- MacAuley D. READER: An acronym to aid critical reading by general practitioners. Br J General Practice. 1994;44(379):83–85.

## Objectively structured clinical examination station scenario

### Objective

This station tests the candidate’s ability to provide care to a patient with eczema.

### Type of station

Integrated consultation.

### Role player

A young man.

### Instructions to the candidate

- You are the family physician overseeing the primary care clinic. The following patient, a homeless person known with atopic eczema, is referred by the clinical nurse practitioner, as he is having an uncontrolled flare-up for the last month.
- Please consult with the patient and manage accordingly.
- Examination findings will be given on request.

## Instructions to the examiner

### Objectives

**FIGURE 3:**
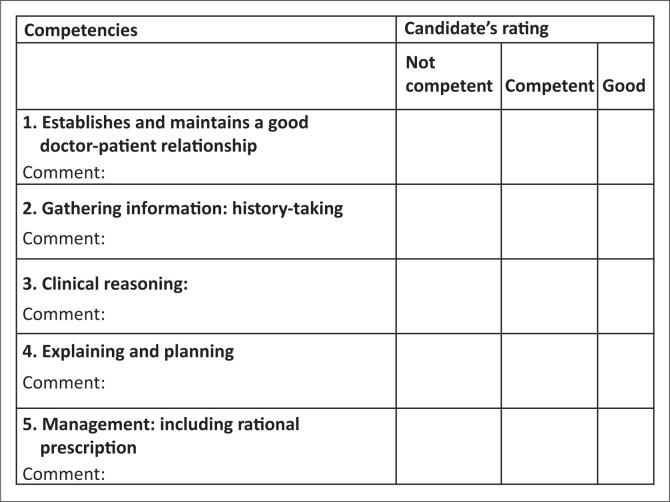
Marking sheet for objectively structured clinical examination station scenario.

- This station tests the candidate’s ability to provide care to a patient with eczema.
- This is an integrated consultation station in which the candidate has 15 min.
- Familiarise yourself with the assessor guidelines, which detail the required responses expected from the candidate.
- No marks are allocated. In the marks sheet (Figure 3), tick off one of the three responses for each of the competencies listed. Make sure you are clear on what the criteria are for judging a candidate’s competence in each area.
- Provide the following information to the candidate when requested: examination findings.
- Please switch off your cell phone.
- Please do not prompt the student.
- Please ensure that the station remains tidy and is reset between candidates.

## Guidelines to examiner

**Working definition of competent performance:** The candidate *effectively completes the task* within the allotted time, in a manner that *maintains patient safety,* even though the execution may not be efficient and well structured.

Establishes a good doctor–patient relationship:

The *competent candidate* acts within the ethical framework (respects autonomy, justice, non-maleficence, beneficence). In addition, the *good candidate* displays empathy and compassion, acknowledging the patient’s discomfort and the anxiety related to ongoing physical symptoms.

Gathering information: history-taking:

The *competent candidate* gathers sufficient information to identify current medical issues [*severe eczema with secondary bacterial infection; associated loss of work productivity*] and identify any ongoing biopsychosocial risks. In addition, the *good candidate* explores the patient’s experience, fears [*fear of permanent disfigurement*] and expectations [*strong steroids*], health-seeking behaviour and identifies opportunities for health promotion [*self-management of flare-up*].

Clinical judgement:

The *competent candidate* uses available evidence to make the correct working diagnosis [*severe eczema with secondary bacterial infection*]. The *good candidate* is able to make a comprehensive three-stage assessment [*as for ‘competent*’, *as well as considering the patient’s fear of disfigurement and potential influence of contextual factors*].

Explaining and planning:

The *competent candidate* clearly explains the working diagnosis [*no jargon; comprehensive; simple language*] and possible interventions. The *good candidate*, in addition, provides a platform for the patient to engage as an equal partner in sharing information and decision-making.

Management:

The *competent candidate* uses current evidence-based guidelines to develop a management plan [*wet wraps; potent steroid cream; aqueous wash and emulsify; consider sedating antihistamine and oral steroids; oral antibiotics*]. In addition, the *good candidate* develops a comprehensive plan using the biopsychosocial approach [*as for ‘competent’ and addresses emotional and contextual factors*].

## Examination findings

- General examination findings: tender axillae and groin lymphadenopathy.
- Ear, nose and throat system findings: no abnormalities.
- Respiratory system findings: no abnormalities.

**FIGURE 4:**
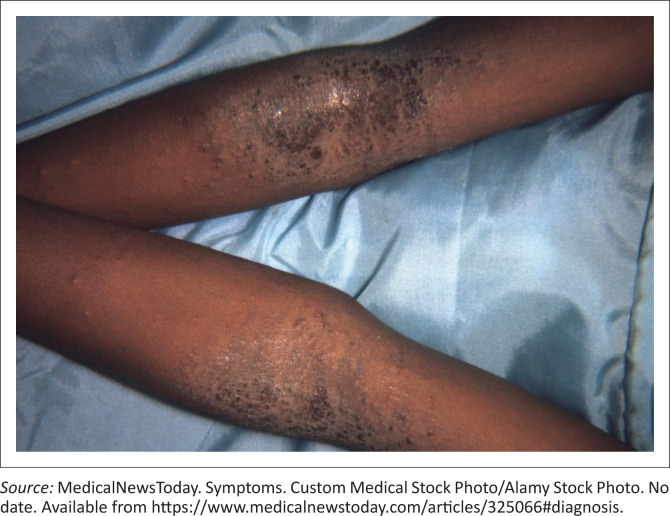
Objectively structured clinical examination clinical scenario.

Skin examination findings (see Figure 4):

- Inflamed, weepy eczema patches on both arms, as well as both legs and thighs.
- Multiple scratch marks and scarring.
- Multiple vesicles on both arms and legs, in various stages of healing with pus exudates.

## Role player instructions

### Appearance and behaviour

A young man.

### Opening statement

‘Doctor, the nurse asked me to come to you. She wants you to change my medication.’

### History

- Open responses: Freely tell the doctor
  ■ You are 23 years old and have had eczema since you were a child.
  ■ You usually get medication, a cream, from the clinic.
  ■ When it flares, they give you stronger creams.
- Closed responses: Only tell the doctor if asked
  ■ Fears: (1) You are very worried that this sickness is going to lead to permanent scarring on your face. (2) You live on the street and so are exposed to the weather all the time. (3) You’re feeling frustrated because this problem just seems to be getting worse all the time.

### Social history

- You have been living on the street since the age of 14, when you ran away from home to get away from an abusive stepfather and alcoholic mother.
- You don’t have a girlfriend and are not sexually active.
- You do petty jobs around the neighbourhood and people give you money or food.

#### If the doctor asks specific questions, offer the following responses:

- Mood: You are OK; not depressed; life on the street is better than at home.
- You have not seen your family in many years.
- You don’t use hard drugs, but smoke cigarettes and dagga sometimes.
- You drink alcohol sometimes but don’t like to be drunk, as this is too dangerous for living on the road.

### Further reading

- National Department of Health, South Africa. Chapter 4.5: Atopic eczema/dermatitis. In: Essential Drugs Programme. Hospital level (adults) Standard Treatment Guidelines. Pretoria: Department of Health; 2019: 142–144.

